# Increased serum levels of macrophage migration inhibitory factor in patients with primary Sjögren's syndrome

**DOI:** 10.1186/ar2182

**Published:** 2007-04-30

**Authors:** Peter Willeke, Markus Gaubitz, Heiko Schotte, Christian Maaser, Wolfram Domschke, Bernhard Schlüter, Heidemarie Becker

**Affiliations:** 1 Department of Medicine B, Muenster University Hospital, Albert Schweitzer Strasse 33, 48129 Muenster, Germany; 2 Institute of Clinical Chemistry and Laboratory Medicine, Muenster University Hospital, Albert Schweitzer Strasse 33, 48129 Muenster, Germany

## Abstract

The objective of this study was to analyse levels of the proinflammatory cytokine macrophage migration inhibitory factor (MIF) in patients with primary Sjögren's syndrome (pSS) and to examine associations of MIF with clinical, serological and immunological variables. MIF was determined by ELISA in the sera of 76 patients with pSS. Further relevant cytokines (IL-1, IL-6, IL-10, IFN-γ and TNF-α) secreted by peripheral blood mononuclear cells (PBMC) were determined by ELISPOT assay. Lymphocytes and monocytes were examined flow-cytometrically for the expression of activation markers. Results were correlated with clinical and laboratory findings as well as with the HLA-DR genotype. Healthy age- and sex-matched volunteers served as controls. We found that MIF was increased in patients with pSS compared with healthy controls (*p *< 0.01). In particular, increased levels of MIF were associated with hypergammaglobulinemia. Further, we found a negative correlation of MIF levels with the number of IL-10-secreting PBMC in pSS patients (*r *= -0.389, *p *< 0.01). Our data indicate that MIF might participate in the pathogenesis of primary Sjögren's syndrome. MIF may contribute to B-cell hyperactivity indicated by hypergammaglobulinemia. The inverse relationship of IL-10 and MIF suggests that IL-10 works as an antagonist of MIF in pSS.

## Introduction

Sjögren's syndrome is an autoimmune disorder characterized by keratoconjunctivitis sicca and xerostomia. Apart from the effects on the lachrymal and salivary glands, various extraglandular manifestations may develop. In addition, an increased risk of lymphoproliferative diseases, especially non-Hodgkin's lymphoma, has been widely described [1]. Focal lymphocytic gland infiltration with upregulated T helper type 1 cytokine expression as well as B-lymphocyte hyperactivity leading to the production of circulating autoantibodies and hypergammaglobulinemia are hallmark characteristics of the disease.

Macrophage migration inhibitory factor (MIF) was discovered in 1966 and initially characterized as a T-cell-derived cytokine that inhibits the migration of macrophages *in vitro *[2,3]. After cloning of MIF in 1989, a much broader range of biological functions has emerged [4]. MIF seems to be a broad-spectrum proinflammatory cytokine with a pivotal role in the regulation of innate and adaptive immune responses [5]. There is increasing evidence for a role of MIF as a proinflammatory cytokine in autoimmune diseases [6]. Serum levels of MIF have been shown to be correlated with the disease activity in several autoimmune disorders including juvenile idiopathic arthritis, rheumatoid arthritis and Wegener's granulomatosis [7-9]. Foote and colleagues [10] recently reported increased MIF levels and a correlation with the disease activity in patients with systemic lupus erythematosus.

Recent findings suggest that MIF might participate in the pathogenesis of other diseases of connective tissue. The present study was designed to elucidate the role of MIF in primary Sjögren's syndrome (pSS).

We examined serum levels of MIF in patients with pSS and the relation of these levels to clinical and laboratory findings. In addition, we analysed associations of MIF concentrations with various cytokines that have been implicated in the pathogenesis of pSS [11,12] as well as with different activation markers on peripheral blood lymphocytes and monocytes.

Moreover, to elucidate whether the production of MIF is influenced by immunogenetic factors we analysed the potential association of MIF levels with distinct HLA-DR genotypes.

## Materials and methods

### Patients and healthy controls

Seventy-six patients with pSS were included in this study. The diagnosis of pSS was based on the American–European Consensus criteria [13]. Patient characteristics and laboratory findings are given in Table 1. None of the participating patients with pSS were on glucocorticoids, but some patients received hydroxychloroquine (*n *= 12) or azathioprine (*n *= 5) as a disease-modifying anti-rheumatic drug. Twenty-eight age- and sex-matched volunteers served as healthy controls.

**Table 1:** Clinical characteristics and laboratory findings of patients with primary Sjögren's syndrome and healthy controls

Parameter	pSS	Controls
Characteristics		
*n*	76	28
Sex (male, female)	3, 73	6, 22
Age^a ^(years)	49.2 ± 13.8	51 ± 11.4
Disease duration^a ^(years)	7.2 ± 4.1	-
Clinical findings		
Conjunctivitis	26 (34)	None
Parotid swelling	22 (28)	None
Arthralgia	51 (67)	None
Myalgia	17 (22)	None
Raynaud's phenomenon	20 (26)	None
Peripheral neuropathy	11 (14)	None
Generalized tendomyopathy	9 (12)	None
Skin involvement	9 (12)	None
Pulmonary involvement	12 (16)	None
Renal involvement	10 (13)	None
Thyroiditis	12 (16)	None
Lymphoma	3 (4)	None
Laboratory findings		
Antinuclear antibodies	76 (100)	Negative
RF (Waaler–Rose test)	60 (79)	Negative
Anti-Ro/SS-A antibodies	69 (91)	Negative
Anti-La/SS-B antibodies	47 (62)	Negative
Hypergammaglobulinemia	57 (75)	Negative
Leukocytopenia	29 (38)	Negative
Anemia	9 (13)	Negative
Thrombopenia	4 (5)	Negative
Low complement C3c	20 (26)	Negative
Low complement C4	12 (16)	Negative

Numbers in parentheses are percentages of the total. pSS, primary Sjögren's syndrome; RF, rheumatoid factor; SS, Sjögren's syndrome. ^a^Mean ± SD.

For analysis of HLA-DR association, 152 healthy sex-matched German Caucasians were used as controls. The study protocol was approved by the local independent ethics committee. Patients and controls gave informed consent to participate in this study.

### Detection of MIF

MIF was detected by enzyme-linked immunoassay as reported previously [14]. In brief, 96-well plates (Nunc GmbH, Wiesbaden, Germany) were coated with mouse anti-human MIF monoclonal antibody (R&D Systems, Wiesbaden, Germany). Non-specific binding sites were blocked by the addition of 250 μl of phosphate-buffered saline containing 1% bovine serum albumin/5% sucrose/0.05% NaN_3 _and incubation for 16 h at 4°C. After plates had been washed three times, recombinant human MIF standards (R&D Systems) and test sera were added to the wells and incubated for 2 h. Biotinylated polyclonal goat anti-human MIF (R&D Systems) was used as the detection antibody and streptavidin–horseradish peroxidase (Jackson/Dianova GmbH, Hamburg, Germany) as the second-step reagent. Colour was developed with 3,3' 5' 5-tetramethylbenzidine (Sigma-Aldrich, Munich, Germany) and absorbance was measured at 450 nm against standard curves. All analyses were performed in duplicate, and mean values were reported. The detection limit of the MIF assay was 0.015 ng/ml.

### ELISPOT analysis and flow cytometry

On samples from 48 consecutive patients with pSS from our cohort, we performed an ELISPOT assay as well as flow cytometric determination of activated lymphocytes and macrophages.

Previously described methods were used for the isolation of peripheral blood mononuclear cells (PBMC) and cell cultures and for the ELISPOT analysis [15].

We analysed the secretion of IL-1, IL-6, IL-10 and TNF-α by unstimulated PBMC. For the detection of IFN-γ, cells were stimulated with 20 μg/ml T-cell mitogen phytohemagglutinin (Endogen, Boston, MA, USA). Spots were automatically counted with an electronic computer-assisted imaging system (Autoimmun Diagnostika GmbH, Strassberg, Germany), which has been shown to be valid and precise [16].

Flow cytometric determination of lymphocyte and monocyte subpopulations was performed by two-colour immunofluorescence analysis on a Coulter XL cytometer (Beckman-Coulter, Krefeld, Germany) as described previously [17]. Activated CD4^+ ^T cells (CD4/CD25, CD4/CD45RO, CD4/CD69, CD4/CD71), activated CD19^+ ^B cells (CD19/CD86) and activated CD14^+ ^monocytes (CD14/HLA-DR) were detected by fluorescein isothiocyanate-labelled and phycoerythrin-labelled monoclonal antibodies (Beckman-Coulter). Results were expressed as the percentage of positive cells.

### Other laboratory parameters

Routine laboratory parameters (namely erythrocyte sedimentation rate, C-reactive protein (CRP), full blood count, total protein and serum electrophoresis) were determined simultaneously. Rheumatoid factor isotypes were analysed by the ELISA technique. We also performed the Waaler–Rose hemagglutination test and the latex fixation test for rheumatoid factor (Dade Behring, Schwalbach, Germany). The levels of IgG antibodies against Ro and La were determined by the ELISA technique (Pharmacia Upjohn, Freiburg, Germany). Serum concentrations of complement levels (C3c and C4) were measured by nephelometry (BN2; Dade-Behring). Low complement levels were defined as C3c levels below 80 mg/dl or C4 levels below 10 mg/dl. Protein electrophoresis was performed with Olympus Hite 320 equipment (Olympus-Diagnostika, Hamburg, Germany). Hypergammaglobulinemia was diagnosed if γ-globulin levels were above 19% in the protein electrophoresis.

### HLA-DR typing

Generic HLA-DR typing was performed by using an enzyme-linked probe hybridization assay (ELPHA; Biotest, Dreieich, Germany). Sequence-specific oligonucleotide probes were used to determine polymorphic sequence motifs. Hybridization between probe and target DNA was detected by a method adapted from the protein ELISA technique.

### Statistics

Data were analysed with the statistical software package SPSS 12.0. Nonparametric tests were used for statistical analysis because a normal distribution of values could not be assumed. The Mann–Whitney *U *test was employed for unpaired samples. The Spearman correlation test was used to correlate laboratory results and clinical data. χ^2 ^and Fisher's exact tests were applied to analyse qualitative variables. *p *< 0.05 was considered significant.

## Results

### Serum MIF levels in patients with pSS and healthy controls

Serum levels of MIF were significantly increased in patients with pSS (median 29.8 ng/ml; range 5.7 to 148 ng/ml) compared with healthy controls (5.7 ng/ml; range 0.015 to 35.3 ng/ml; *p *< 0.01). No significant differences of MIF levels were found between patients with pSS receiving therapy with disease-modifying anti-rheumatic drugs and those not receiving it, nor did we observe any differences of MIF levels between patients taking hydroxychloroquine or azathioprine. There was no association of MIF with disease duration or age of patients.

### Association of MIF levels with laboratory and clinical features

**Figure 1:**
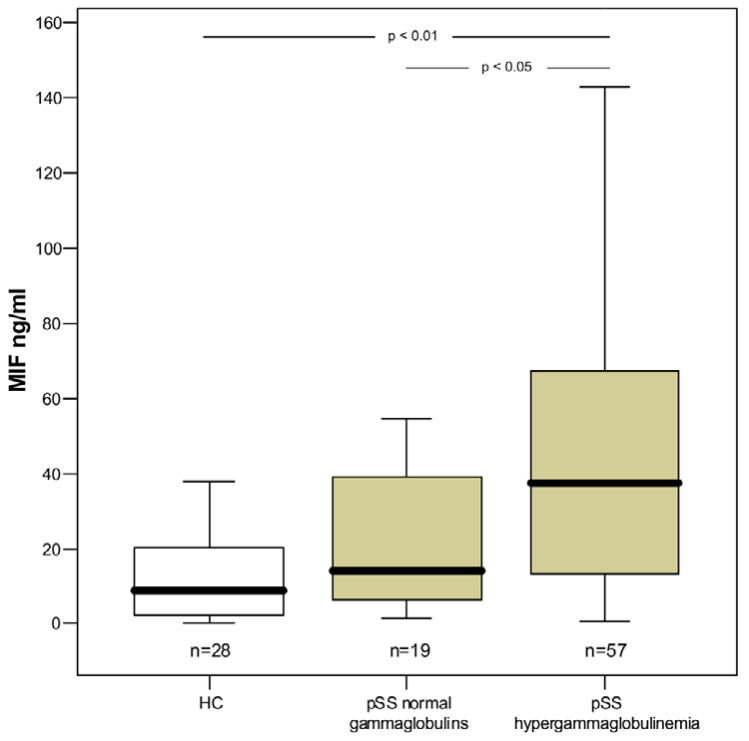
Serum levels of macrophage migration inhibitory factor (MIF). Data of the box plots are shown as medians and 25th and 75th centiles for healthy controls (HC) as well as for primary patients with Sjögren's syndrome (pSS) with normal γ-globulins and with hypergammaglobulinemia.

Patients with hypergammaglobulinemia (*n *= 57) had significantly increased levels of MIF compared with healthy controls (*p *< 0.01) and compared with patients with pSS with normal γ-globulins (*p *< 0.05; Figure 1). Correspondingly, the percentage of γ-globulins also correlated with MIF serum levels (*r *= 0.278, *p *< 0.05).

There were no associations of MIF levels with anti-Ro or anti-La antibody titers, rheumatoid factor isotypes or other laboratory findings listed in Table 1. None of the patients with pSS had an increased level of CRP.

There was a tendency for increased numbers of IL-10-secreting PBMC in patients with pSS (*p *< 0.066, data not shown). Numbers of PBMC secreting IL-1, IL-6, IFN-γ or TNF-α did not differ significantly from those in healthy controls (data not shown).

**Figure 2:**
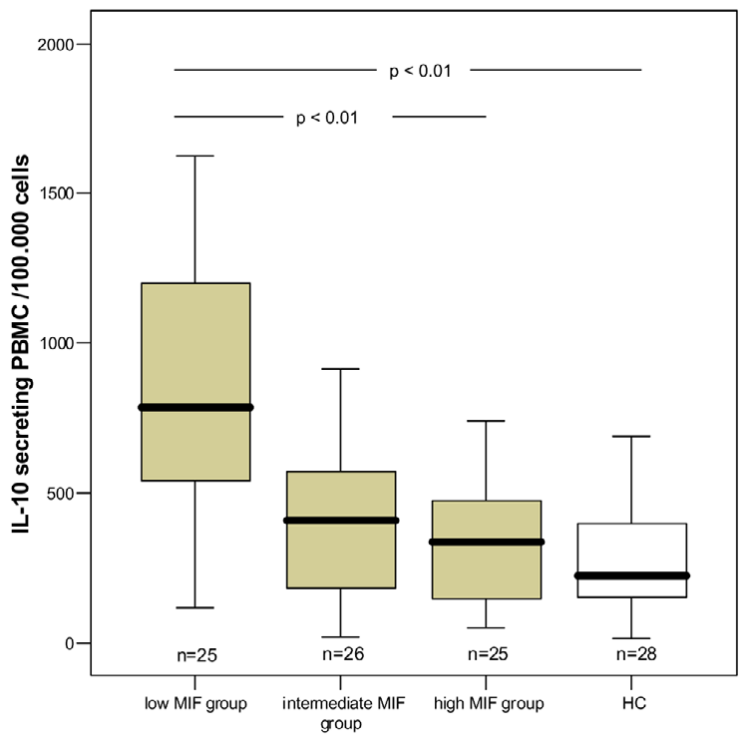
IL-10-secreting peripheral blood mononuclear cells (PBMC) in patients with primary Sjögren's syndrome and healthy controls (HC). Data of Sjögren's syndrome patients were divided into a low-MIF group (0 to 33%), an intermediate-MIF group (more than 33 to 66%) and a high-MIF group (more than 66%). The number of IL-10-secreting PBMC in the low-MIF group was significantly increased compared with the high-MIF group or healthy controls.

We found a negative correlation of MIF levels with the number of IL-10-secreting PBMC (*r *= 0.389, *p *< 0.01). Patients with low MIF levels had a significantly increased number of IL-10-secreting PBMC compared with patients with high MIF levels and compared with healthy controls (*p *< 0.01; Figure 2). In contrast, there were no significant associations of MIF with numbers of PBMC secreting IL-1, IL-6, IFN-γ or TNF-α.

The percentage of CD4/CD71^+ ^T cells was significantly increased in patients with pSS compared with healthy controls (*p *< 0.05, data not shown) but there were no significant correlations of MIF levels with the expression of different activation markers on CD4^+ ^T cells (CD4/CD25, CD4/CD45RO, CD4/CD69, CD4/CD71), CD19^+ ^B cells (CD19/CD86) or CD14^+ ^monocytes (CD14/HLA-DR). No significant correlations in the absolute numbers of these cell populations were observed.

We found no associations of MIF levels with different glandular or extraglandular manifestations listed in Table 1. In the three patients with a previous B-cell lymphoma the MIF level was significantly increased compared with that in healthy controls (*p *< 0.01) but did not differ significantly from that of other patients with pSS without lymphoma.

### HLA-DR associations

The prevalence of HLA-DR3 was increased in patients with pSS compared with healthy controls (34.3% versus 12.5%; *p *< 0.01, data not shown). There was no association of MIF levels with different HLA-DR genotypes.

## Discussion

Our data show significantly increased serum levels of MIF in patients with pSS, especially in those with increased γ-globulins. Hypergammaglobulinemia has been linked with the extent of histopathological salivary gland abnormalities and has been proposed as an activation marker in pSS [18,19]. An increased production of γ-globulins results from polyclonal B-cell hyperactivity [20]. MIF can provide signals for B cells to proliferate [21]. It has been shown that neutralization of MIF significantly inhibits antibody production *in vivo *[22]. Increased production of MIF might therefore contribute to hypergammaglobulinemia and possibly reflects disease activity of pSS.

MIF has been associated with various autoimmune diseases [7-10]. The induction and regulation of MIF in autoimmune diseases is not well characterized [23]. It has been shown that low concentrations of glucocorticoids induce MIF production from macrophages; this could be part of a counter-regulatory system that functions to control immune responses [24]. Previous results showing increased levels of MIF in patients with systemic lupus erythematosus could partly be explained by corticosteroid use [10]. However, because our patients with pSS did not receive any glucocorticoids, the increased MIF levels could not be explained by this argument.

MIF has been shown to be increased in acute inflammation and a correlation with CRP concentrations has been described [8,25]. As the acute-phase reactant CRP was not elevated in our cohort, the increased MIF levels in patients with pSS cannot be explained by differences in the extent of acute-phase response. Hence, specific mechanisms of MIF induction in pSS remain to be elucidated.

There was an increased prevalence of HLA-DR3 in our cohort of patients with pSS, confirming previous findings [26]. Antibodies against Ro and La have been shown to be associated with HLA-DR3, possibly as a result of HLA haplotype-dependent differences in presentation of autoantigens and subsequent stimulation of the immune response [26]. We detected no associations of MIF levels with the HLA-DR genotype, suggesting that HLA-DR polymorphism does not have a major role in the generation of MIF.

We found a negative correlation of MIF with IL-10-secreting PBMC. In addition, we observed a tendency towards an increased number of IL-10-secreting PBMC in our cohort, as reported previously [27]. It has been shown *in vitro *that IL-10 inhibits MIF synthesis [28]. Moreover, neutralization of MIF leads to an increase of IL-10 production in an animal model [29]. IL-10 has been described as a potent macrophage deactivator that inhibits cytokine production by activated macrophages [30]. Our data might indicate downregulation of MIF by IL-10 *in vivo *and suggest that IL-10 and MIF are part of a negative regulatory circuit. It might be assumed that IL-10 counteracts MIF-induced inflammatory processes such as activation of macrophages, as reported previously [28].

We found no association of MIF with other cytokine-secreting PBMC in our patients. It has been shown that MIF can upregulate proinflammatory cytokines including TNF-α *in vitro *[31]. However, other authors have not identified any TNF-inducing effect of MIF on PBMC [32]. Recently it has been shown that MIF alone is not sufficient to induce cytokine expression: co-stimulators such as lipopolysaccaride are necessary to induce the secretion of TNF-α and IL-1 [33]. This suggests that MIF may act to modulate and amplify the response to lipopolysaccharide in sepsis. In pSS, MIF apparently has no inducing effect on TNF-α or other proinflammatory cytokines analysed, presumably because of a lack of such co-stimulatory factors.

The percentage of CD4/CD71^+ ^T cells was significantly increased in patients with pSS compared with healthy controls, as reported previously [34]. We did not find an association of MIF with various activation markers on T helper cells, B cells or macrophages, although MIF has been identified as an activator of B and T cells as well as macrophages [4,21,22]. Recently it has been suggested that MIF is a critical effector of organ injury in systemic lupus erythematosus in the absence of major changes in T-cell and B-cell markers or alterations in autoantibody production [35]. Most probably this observation holds also true for pSS.

MIF has no homology with any other proinflammatory cytokine, and the mechanisms by which MIF exerts its biological effects are not yet fully understood [33]. It is possible that MIF mediates organ injury directly, because it has been shown that MIF induces the production of matrix metalloproteinase-9 [36], which has been implicated in the pathogenesis of pSS [37]. MIF can stimulate the inducible nitric oxide synthase and increase the production of nitric oxide, which can directly mediate cell injury [38]. It has been suggested that nitric oxide contributes to inflammatory damage and acinar cell atrophy in Sjögren's syndrome [39].

Our three patients with B-cell lymphoma had increased MIF levels compared with healthy controls, but there were no significant differences from patients with pSS without B-cell lymphoma.

Patients with pSS are at increased risk of developing B-cell non-Hodgkin's lymphoma [1]. It has been suggested that MIF provides a link between inflammation and tumorigenesis [21,40].

MIF expression is increased in sporadic human colorectal adenomas [41]. MIF has been shown to decrease the tumor suppressor activity of p53 and to upregulate Bcl-2 expression [42], which has been suggested to be important in B-cell monoclonal proliferation and malignant transformation in pSS [43]. A deficiency of p53 tumor suppressor activity is associated with the development of low-grade mucosa-associated lymphoid tissue lymphoma [44]. It has been shown in a lymphoma mouse model that loss of MIF markedly delays the onset of B-cell lymphoma development *in vivo *[45].

## Conclusion

This study provides the first *in vivo *evidence for a potential role of MIF in pSS. Additional investigation is required to substantiate the association of MIF with disease activity and the development of lymphomas. Eventually, targeting of MIF may offer therapeutic options in this autoimmune disease.

## Abbreviations

CRP = C-reactive protein; ELISA = enzyme-linked immunosorbent assay; IL = interleukin; IFN = interferon; MIF = macrophage migration inhibitory factor; PBMC = peripheral blood mononuclear cells; pSS = primary Sjögren's syndrome; TNF = tumor necrosis factor.

## Competing interests

The authors declare that they have no competing interests.

## Authors' contributions

PW participated in the data analysis and the design of the study, and drafted the manuscript. MG, HS and HB helped with data collection, patient recruitment and the design of the study. MG, HS, WD, CM and HB helped in editing the manuscript. CM provided technical help for the MIF analysis. BS participated in the design and helped in the statistical analysis. All authors read and approved the final manuscript.
